# Mutual synchronization of eyeblinks between dogs/cats and humans

**DOI:** 10.1093/cz/zoab045

**Published:** 2021-06-04

**Authors:** Hikari Koyasu, Risa Goto, Saho Takagi, Miho Nagasawa, Tamami Nakano, Takefumi Kikusui

**Affiliations:** Laboratory of Human-Animal Interaction and Reciprocity, Azabu University, 1-17-71 Fuchinobe, Chuo-ku, Sagamihara-shi, Kanagawa, 252-5201, Japan; Japan Society for the Promotion of Science, 5-3-1 Kojimachi, Chiyoda-ku, Tokyo, 102-0083, Japan; Laboratory of Human-Animal Interaction and Reciprocity, Azabu University, 1-17-71 Fuchinobe, Chuo-ku, Sagamihara-shi, Kanagawa, 252-5201, Japan; Laboratory of Human-Animal Interaction and Reciprocity, Azabu University, 1-17-71 Fuchinobe, Chuo-ku, Sagamihara-shi, Kanagawa, 252-5201, Japan; Japan Society for the Promotion of Science, 5-3-1 Kojimachi, Chiyoda-ku, Tokyo, 102-0083, Japan; Laboratory of Human-Animal Interaction and Reciprocity, Azabu University, 1-17-71 Fuchinobe, Chuo-ku, Sagamihara-shi, Kanagawa, 252-5201, Japan; Graduate School of Frontiers and Biosciences, Osaka University, 1-3 Yamadaoka, Suita, Osaka, 565-0871, Japan; Laboratory of Human-Animal Interaction and Reciprocity, Azabu University, 1-17-71 Fuchinobe, Chuo-ku, Sagamihara-shi, Kanagawa, 252-5201, Japan

**Keywords:** blink, cat, communication, dog, synchronization

In humans *Homo sapiens*, eyeblinks play an important role in communication. Blinks are synchronized between 2 individuals during conversation, with shared breaks between contexts, suggesting that blink synchronization can facilitate the sharing of a rhythm during communication between 2 individuals. In the case of individuals with autism spectrum disorder, whose major symptom is impaired communication, there is no blink synchronization (Nakano and Kitazawa 2010; Nakano et al. 2011). Therefore, blink synchronization potentially occurs in 2 individuals who can communicate effectively. In addition, blink synchronization is correlated with the degree of synchronization of activity in the right inferior frontal gyrus of the brain (Koike et al. 2016). It suggests that being in the same physiological state as one’s partner based on similar brain activity could facilitate mutual understanding. Synchronization of blinks leads to effective communication in humans. Recently, it has been demonstrated that blinks have a social function in nonhuman primates (Tada et al. 2013; Ballesta et al. 2016). It has also been suggested that dogs *Canis familiaris* and cats *Felis silvestris catus* use blinks as communication signals with humans (Kuhne et al. 2012; Koyasu and Nagasawa 2019). For example, Humphrey et al. reported that the approaching behavior of cats increased when the human observer blinked (Humphrey et al. 2020). While they did not report blink synchronization in the behavioral test, it is possible that synchronized blinks between cats and humans facilitate approach behavior. In this study, the temporal relationships of blinks between dogs/cats and humans were analyzed. The humans had 5 gaze interactions for 1 min with a dog or a cat. The blinks of dogs/cats and humans were recorded during gaze interactions, and we investigated when the human blinked before and after the dog/cat blinked. The effects of affiliation were examined to explore if blink synchronization was associated with affiliation between humans and dogs/cats. We predicted that blink synchronization would be observed between dogs/cats and their owners, who could communicate effectively. Twenty-six dogs and 24 cats participated in this study. We excluded cases that lacked proper recordings, including cases in which more than 30% of the experimental footage did not capture the eyes of dogs or cats or humans, and cases where experiments were interrupted due to stress states in the dogs or cats. We used 7 pairs of dogs and humans and 10 pairs of cats and humans for the analyses (Supplementary Table S1). Detailed methods are described in Supplementary Materials.

The blink rate of humans was 28.994 ± 12.814 bpm (average ± *SD*) during the tests. The dog’s blink rate was 6.529 ± 3.752 bpm and the cat’s blink rate was 4.103 ± 2.695 bpm (Supplementary Table S2). As the results of blink temporal analyses, the frequency of human blinks was higher at certain time windows before and after the dogs or cats blinked (Figure 1). Regarding human blink frequency after dogs/cats blinked in each figure (indicating positive time window), the owners’ blink frequency was high, at 0.00–0.25 s, in the case of dogs (Figure 1A; *P = *0.001). Similarly, in the case of cats, the owners’ blink frequencies were high, at 0.00–0.25 s, 0.25–0.50 s, and 0.50–0.75 s (Figure 1C; *P < *0.001, *P = *0.002, *P < *0.001, respectively). Additionally, strangers’ blink frequencies were high, at 0.50–0.75 s, in the case of dogs (Figure 1B; *P = *0.014). In the case of cats, strangers’ blink frequencies were high, at 1.00–1.25 s (Figure 1D; *P < *0.001). Owner blink frequencies were high immediately following the blinks of both dogs and cats, but strangers took longer to respond. Regarding the human blink frequency before dogs/cats blinked in each figure (indicating negative time window), both owner and stranger blink frequencies were high, at 0.75–1.00 s before the dogs’ blinks (Figure 1A and B; *P = *0.005, *P = *0.001, respectively). Furthermore, both owner and stranger blink frequencies were high, at 1.00–1.25 s, before the cats’ blinks (Figure 1C and D; *P < *0.001, *P < *0.001, respectively). Taking human blinks before the dogs/cats blinked as dogs/cats' blinks after the humans blinked, dogs and cats blinked ∼1 s after the human blinked.

**Figure 1.**
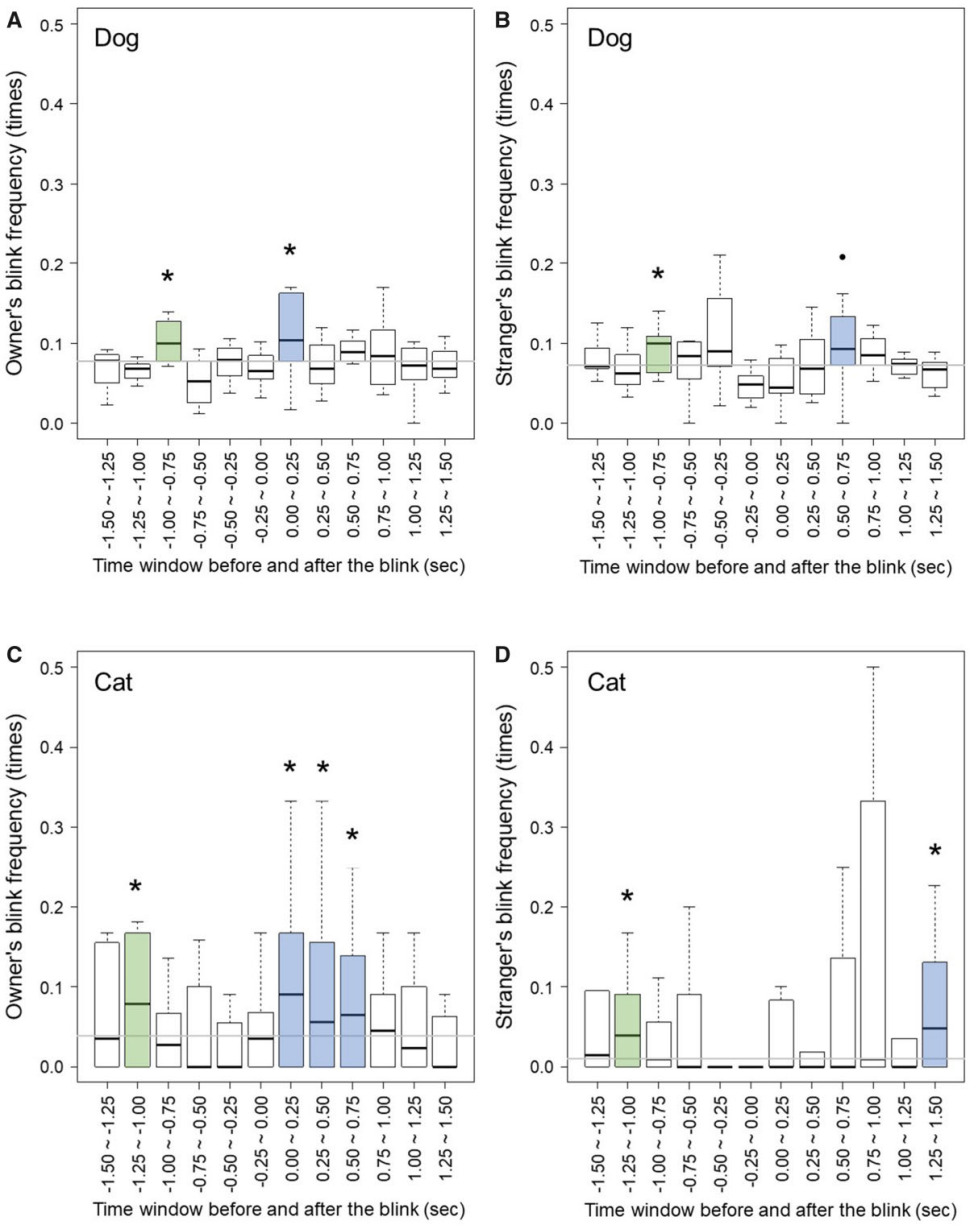
Human blink frequency before and after the dogs and cats blink. (**A** and **C**) The owner’s blink frequency. (**B** and **D**) The stranger’s blink frequency. The horizontal axis indicates the time windows before (negative) and after (positive) dogs (A and B) and cats' (C and D) blink onset. The vertical axis indicates the human blink frequency included in the window of each time. The time window shown in minus is the frequency of human blinks that occurred before the blinks of dogs and cats. The positive time window is the frequency of human blinking that occurred after the blinks of dogs and cats. The asterisks indicate that human blink frequency was significantly higher than chance level. The circle indicates a significant tendency.

To examine whether human blink frequencies influence dogs/cat eyeblink frequencies, we compared the frequencies of eyeblink between humans and dogs/cats (Figure 2). There was no correlation between blink rates of humans and dogs/cats through the whole test (rs = 0.282, *P = *0.146; rs = −0.01, *P = *0.961, respectively). Similar results were found when examining mutual gaze and 1-sided gaze independently (mutual gaze: human–dog, rs = 0.503, *P = *0.067, Figure 2A; human–cat, rs = 0.038, *P = *0.875, Figure 2B; 1-sided gaze: human–dog, rs = 0.072, *P = *0.805, Figure 2A; human–cat: rs = 0.053, *P = *0.826, Figure 2B).

**Figure 2.**
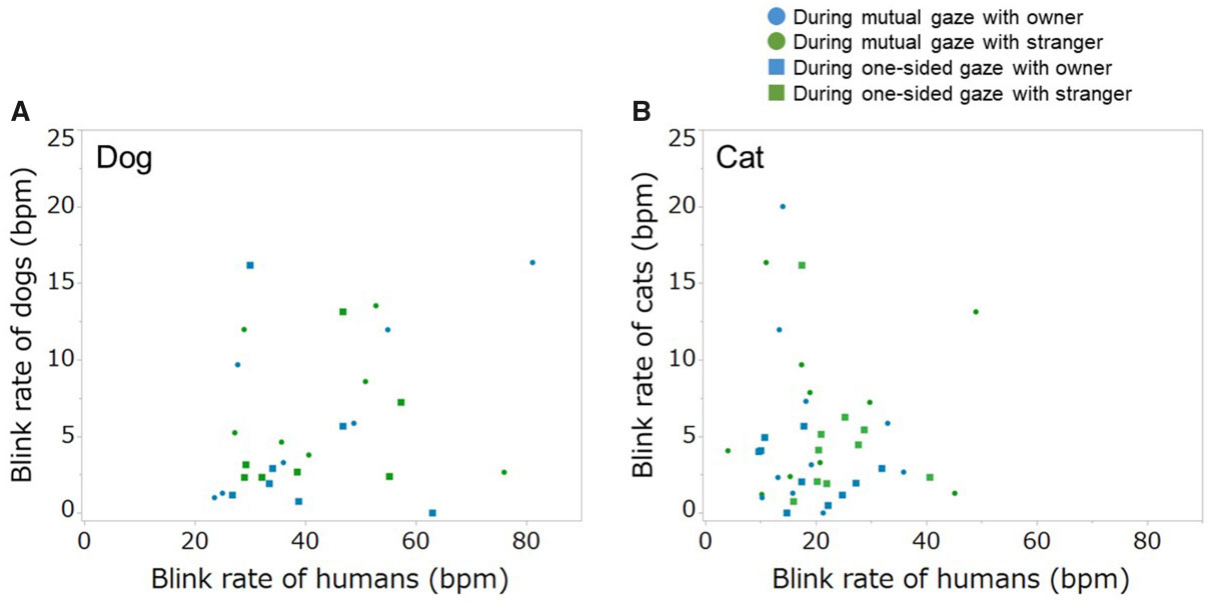
Correlation of blink rate between humans and dogs/cats. Blink rates during mutual gaze (circle) and 1-side gaze (square) between dogs/cats and their owners (blue) and between dogs/cats and strangers (green). (**A**) Correlation between the human and dog blink rate, (**B**) correlation between human and cat blink rate. The blink rate of dogs/cats with owners and the blink rate of owners are shown in blue, and the blink rate of dogs/cats with strangers and the blink rate of strangers are shown in green.

In summary, although the time lag varied, mutual synchronization of blinks existed between humans and dogs/cats; with humans blinking after dogs/cats blinked, and dogs/cats blinking after humans did (Figure 1). In addition, there was no correlation between human blink rates and dog/cat blink rates (Figure 2). If the dogs and cats increased the rates of blinking depending on the human blink, there would be a positive correlation between human blink rates and dog/cat blink rates. This indicated that blink synchronization in this study was not caused by increased blink rates, but by adjustment of the timing of spontaneous blinks to the timing of the partner. Most of the time lags observed in this study were categorized as mimicry, in synchronization. Mimicry is performing the same behavior but not immediately. Considering that the blink synchronization leads to the sharing of a communication rhythm in humans, automatic mimicry of blinks may lead to the sharing of a communication rhythm between dogs/cats and humans as well. Owners, unlike the strangers, had short time lags after their dogs or cats, and their dogs/cats blinked ∼1 s after the humans blinked, regardless of affiliation. The socially closer humans are, the shorter the time lag in synchronization of their yawns (Norscia and Palagi 2011). Similar to that the time lag of yawn synchronization is influenced by affinity, affiliation to dogs/cats by ownership could lead to relatively short time lags in owners’ blinks. Regarding the lack of influence of affiliation on blink time lags of dogs and cats following human blinking, the dogs and cats perhaps did not distinguish between the blinks of their owners and those of strangers, or could communicate effectively with both owners and strangers. Numerous investigations have shown that dogs and cats distinguish their owners from strangers; however, the magnitude of the effect is context dependent (e.g., Horn et al. 2013; Potter and Mills 2015). Observation of blink synchronization under various social contexts could reveal the influence of human affiliation to a dog or a cat. Furthermore, in the future, researchers should explore how blink synchronization influences the relationship. For example, behavioral changes that occur after blink synchronization could reveal the potential function of blink synchronization; greater synchronization between dogs/cats and humans could indicate greater affiliation.

## Ethics Approval

All (animal and human) protocols were carried out in accordance with relevant guidelines and regulations. All experimental procedures for dogs and cats were approved by the Animal Ethics Committee of Azabu University (#180410-1). Experimental procedures for human participants were approved by the Ethical Committee for Medical and Health Research Involving Human Subjects of Azabu University (#052).

## Funding

This work was supported by the Japan Society for the Promotion of Science, and Grants-in-Aid for Scientific Research from the Ministry of Education, Culture, Sports, Science, and Technology of Japan (#20J14760 to H.K. #18H02489, #19K22823, #21H03333 to M.N., and #19H00972 to T.K.).

## Conflict of Interest

The authors declare that they have no conflict of interest.

## Authors’ Contributions

H.K. planned and conducted the experiments and wrote the article. R.G. analyzed the dog data. S.T. contacted the cat’s owners and collected the data. M.N., T.N., and T.K. discussed the data and wrote the article.

## Supplementary Material

Supplementary material can be found at https://academic.oup.com/cz.

## Supplementary Material

zoab045_Supplementary_Data
