# Supplementary material for: Mutual synchronization of eyeblinks between dogs/cats and humans
Source: Curr Zool. 2021 Jun 4;68(2):229–32. doi: 10.1093/cz/zoab045 (PMC8962689; doi:10.1093/cz/zoab045)
Supplement: zoab045_Supplementary_Data [file zoab045_supplementary_data.zip › zoab045-suppl_data/Supplemental_Experimental_Procedures.pdf]

Mutual Synchronization of Eyeblinks between Dogs/Cats and Humans

Hikari Koyasu[a, b], Risa Goto[a], Saho Takagi[a, b], Miho Nagasawa[a], Tamami Nakano[c], Takefumi Kikusui[a,*]

[a]Laboratory of Human-Animal Interaction and Reciprocity, Azabu University, 1-17-71 Fuchinobe, Chuo-ku, Sagamihara-shi, Kanagawa 252-5201, Japan

[b]Japan Society for the Promotion of Science, 5-3-1 Kojimachi, Chiyoda-ku, Tokyo 102-0083, Japan

[c]Graduate School of Frontiers and Biosciences, Osaka University, 1-3 Yamadaoka, Suita, Osaka 565-0871, Japan

*Address correspondence to Takefumi Kikusui. Email: kikusui@azabu-u.ac.jp

Handling editor: Zhi-Yun Jia (贾志云)

Received on 16 March 2021; accepted on 28 May 2021

Keywords: blink, synchronization, communication, cat, dog

Supplemental Experimental Procedures

For dogs, the tests were performed in the laboratory at Azabu University, Japan. The room was 2.5 meters × 5.0 meters, and was partitioned in half, with a dog on one side and a human on the other side. There was a 25 cm clear window in the center of the partition where humans and dogs could make eye contact. For cats, the tests took place in a room of the owner's house. While the cat was calm in their carrier bag or in their bed, the owner or stranger sat in front of the cat and conducted the experiment. Two video cameras were used to record behavior (HX-A1H, Panasonic Corporation, Osaka, Japan; HDR-AS50, Sony Corporation, Tokyo, Japan). The sampling rate was 60 fps. Kinovea (version 0.8.15) was used for recording the blinks of humans and dogs/cats, and BORIS (version 7.10.2) was used for recording the duration that dogs and cats looked at humans. Blinks were defined as a movement of eyelids taking less than two seconds. It included incomplete blinks, such as half blinks and one-eye blinks. The number of humans acting as strangers was 27. Because the dog experiments were conducted at the laboratory, 26 college students participated as strangers. However, as the cat experiments were conducted in each owner's house, only one stranger participated. The sex of the stranger was the same as that of the owner. In the current study, the influence of sex and age of either dogs/cats or humans was not take into consideration because of small sample size.

The timing of the blinks of humans before and after the blinks of dogs/cats was analyzed. Human blink frequencies were counted three-seconds before and after the blinks of dogs/cats, using the time series of blinks for the dogs/cats as the basis for a series of three-second analysis windows (1.50 s before and 1.50 s after blink onset). A blink frequency histogram was constructed for each subject by dividing these 3 s into 12 equal windows (0.25 s) and used in the subsequent analyses. When a human blink was counted more than once in different windows, the duplicated count was adjusted by dividing each blink by the total number of blinks. Subsequently, the frequencies of human blinks divided by the times of dog and cat blinks were used to calculate the human blink frequency per dog or cat blink. A binomial test was used to determine whether the actual human blink frequency was higher than the chance level. The chance level

was determined by dividing the sum of the median human blink frequencies occurring in each time window before and after the dog/cat blink by 12 (the number of time windows). Because of the repetitive analyses, the false discovery rate was adjusted using the Benjamini-Hochberg procedure. Note that we did not conduct the analysis based on the timing of human blinking because the frequency of blinks of dogs/cats (especially cats) is very different from that of humans. We analyzed the human blink frequency that occurred before the dogs/cats blinked instead.

In addition, blink rates were calculated as the frequencies of blinks per minute during the mutual gaze and one-sided gaze. The time when the dogs or cats looked at the human was defined as mutual gaze, and the time when the dogs or cats did not look at the humans was defined as the one-sided gaze of humans. Blink rate during mutual gaze was determined as the number of blinks during mutual gaze divided by the duration of mutual gaze, and blink rate during one-sided gaze was determined as the number of blinks during one-sided gaze divided by the duration of one-sided gaze. Spearman's rank correlation coefficients were determined to assess the relationship between blink rates of humans and dogs/cats. Statistical analysis software R (version 3.5.2) was used.

All (Animal and Human experiments) protocols were carried out in accordance with relevant guidelines and regulations. All experimental procedures for dogs and cats were approved by the Animal Ethics Committee of Azabu University (#180410-1). Experimental procedures for human participants were approved by the Ethical Committee for Medical and Health Research Involving Human Subjects of Azabu University (#052). All participants gave written informed consent, approved by the ethical committee, prior to the experiments.

Table S1. Subject Information

The information of the dogs and cats that participated in the experiment are shown. Individuals with gray shaded were excluded from the analysis.

ID	Species	Sex	Age	Breed	
D1	Dog	Female	6	Mix	
D2	Dog	Male	5	Pomeranian	More than 30% of time was not captured human eyes
D3	Dog	Male	11	Dalmatian	
D4	Dog	Female	13	Dalmatian	
D5	Dog	Female	10	Toy pooble	Excluded for the condition of the dog
D6	Dog	Female	10	Shih Tzu	Human blinks are extremely low
D7	Dog	Female	5	Border collie	More than 30% of time was not captured dog's eyes
D8	Dog	Male	3	Border collie	More than 30% of time was not captured dog's eyes
D9	Dog	Male	12	Shiba	No blinks during the test
D10	Dog	Male	6	Toy pooble	Excluded for the condition of the dog
D11	Dog	Female	8	Chihuahua	More than 30% of time was not captured human eyes
D12	Dog	Female	1	Mix	More than 30% of time was not captured dog's eyes
D13	Dog	Male	8	Weimaraner	More than 30% of time was not captured dog's eyes
D14	Dog	Male	4	Golden retriever	More than 30% of time was not captured dog's eyes
D15	Dog	Male	8	Miniature Bull Terrier	Different frame rates
D16	Dog	Female	3	Chihuahua	More than 30% of time was not captured dog's eyes
D17	Dog	Male	9	Toypoodle	No video of the owner
D18	Dog	Male	4	Golden retriever	Different frame rates
D19	Dog	Male	2	Toypoodle	
D20	Dog	Female	1	Toypoodle	
D21	Dog	Female	14	Jack russel terrier	Different frame rates
D22	Dog	Female	14	Jack russel terrier	No video of the owner
D23	Dog	Male	-	Mix	More than 30% of time was not captured dog's eyes
D24	Dog	Male	-	Mix	
D25	Dog	Male	-	Mix	More than 30% of time was not captured dog's eyes
D26	Dog	Male	-	Mix	
C1	Cat	Female	10	Mix	More than 30% of time was not captured cat's eyes
C2	Cat	Male	3	Ragamuffin	Different frame rates

C3	Cat	Female	3	American Shorthair	
C4	Cat	Female	7	Mix	
C5	Cat	Male	9	Mix	More than 30% of time was not captured cat's eyes
C6	Cat	Male	3	American Curl	More than 30% of time was not captured cat's eyes
C7	Cat	Male	4	Persian	Test interruptions due to cat conditions
C8	Cat	Male	2	Mix	
C9	Cat	Male	1	Mix	
C10	Cat	Male	1	Mix	Test interruptions due to cat conditions
C11	Cat	Male	8	American Shorthair	Excluded for unification of test conditions
C12	Cat	Male	5	Ocicat	Excluded for unification of test conditions
C13	Cat	Male	20	Mix	Test interruptions due to cat conditions
C14	Cat	Female	4	Mix	
C15	Cat	Male	9	Mix	
C16	Cat	Female	9	Mix	More than 30% of time was not captured cat's eyes
C17	Cat	Male	15	Russian Blue	
C18	Cat	Male	3	Scottish Fold	
C19	Cat	Male	5	Persian	Excluded for unification of test conditions
C20	Cat	Male	2	Mix	More than 30% of time was not captured cat's eyes
C21	Cat	Male	6	British Shorthair	
C22	Cat	Male	3	Mix	Test interruptions due to cat conditions
C23	Cat	Female	3	Mix	
C24	Cat	Male	14	Mix	No blinks during the test

Table S2. Blink rates of humans, dogs and cats

The blink rates of humans (A), dogs (B), and cats(C) are shown. The IDs correspond to the dogs and cats information in TableS1. The "With Owner/Stranger" column in the table of "Dogs' blink rates (B)" and "Cats' blink rates (C)" describe whether the dogs/cats participated in the test with the Owner or the Stranger.

(A) Human blink rates

ID	Owner/Stranger	Sex	During mutual gaze	During One-sided gaze	Whole test
D1	Owner	Female	52.813	55.276	54.801
D1	Stranger	Female	48.822	26.839	29.000
D3	Owner	Female	35.724	38.588	36.600
D3	Stranger	Female	81.184	30.041	50.200
D4	Owner	Female	27.262	32.176	29.400
D4	Stranger	Female	54.971	63.068	60.800
D19	Owner	Male	76.035	29.284	37.400
D19	Stranger	Male	27.790	33.518	28.200
D20	Owner	Female	40.662	46.855	43.200
D20	Stranger	Female	23.593	32.715	28.333
D24	Owner	Male	28.947	29.014	29.000
D24	Stranger	Male	25.032	34.119	32.667
D26	Owner	Female	50.926	57.365	55.500
D26	Stranger	Female	36.062	38.842	38.626
C3	Owner	Female	45.167	40.662	41.400
C3	Stranger	Female	21.333	24.812	24.420
C4	Owner	Female	18.935	17.474	18.400
C4	Stranger	Female	18.211	14.768	17.667
C8	Owner	Female	10.210	21.979	20.250
C8	Stranger	Female	14.024	17.848	17.667
C9	Owner	Female	4.078	15.972	15.000
C9	Stranger	Female	33.014	31.968	32.000
C14	Owner	Female	10.993	20.516	19.000
C14	Stranger	Female	13.386	10.746	11.667
C15	Owner	Female	17.413	20.967	20.600
C15	Stranger	Female	10.267	9.680	10.000
C17	Owner	Female	20.767	20.260	20.333
C17	Stranger	Female	15.821	17.445	17.000
C18	Owner	Female	29.775	28.771	29.000
C18	Stranger	Female	13.138	22.231	22.000
C21	Owner	Female	15.348	27.708	26.500
C21	Stranger	Female	19.183	10.047	14.333
C23	Owner	Female	48.992	25.286	26.836
C23	Stranger	Female	35.899	27.280	28.000

(B) Dogs' blink rates

ID	With Owner/Stranger	During mutual gaze	During one-sided gaze	Whole test
D1	Owner	13.524	7.074	8.600
D1	Stranger	12.027	3.508	6.200
D3	Owner	4.625	6.042	5.000
D3	Stranger	3.297	4.068	3.600
D4	Owner	5.234	9.703	6.800
D4	Stranger	10.283	7.060	8.000
D19	Owner	2.656	5.075	3.800
D19	Stranger	5.343	0.000	5.200
D20	Owner	3.784	0.547	2.600
D20	Stranger	1.157	0.611	0.800
D24	Owner	8.575	9.206	9.000
D24	Stranger	2.554	5.208	5.000
D26	Owner	11.973	11.742	11.800
D26	Stranger	13.932	15.400	15.000

(C) Cats' blink rates

ID	With Owner/Stranger	During mutual gaze	During one-sided gaze	Whole test
C3	Owner	1.2867	2.3218	2.000
C3	Stranger	0.0000	1.1685	1.000
C4	Owner	7.8586	16.1635	9.400
C4	Stranger	7.2883	0.0000	6.333
C8	Owner	1.2110	1.9165	1.800
C8	Stranger	19.9990	5.6604	7.333
C9	Owner	4.0630	0.7471	1.400
C9	Stranger	5.8519	2.8991	3.000
C14	Owner	16.3451	4.1039	6.800
C14	Stranger	11.9571	4.9203	7.667
C15	Owner	9.6740	5.1303	5.600
C15	Stranger	0.9999	4.0004	2.200
C17	Owner	3.2874	2.0494	2.200
C17	Stranger	1.2885	2.0303	1.800
C18	Owner	7.2181	5.4199	6.333
C18	Stranger	2.3177	0.4834	0.800
C21	Owner	2.3763	4.4443	3.400
C21	Stranger	3.1455	4.0705	3.600
C23	Owner	13.1265	6.2470	7.400
C23	Stranger	2.6676	1.9459	2.000
