# Supplementary material for: Mutual synchronization of eyeblinks between dogs/cats and humans
Source: Curr Zool. 2021 Jun 4;68(2):229–32. doi: 10.1093/cz/zoab045 (PMC8962689; doi:10.1093/cz/zoab045)
Supplement: zoab045_Supplementary_Data [file zoab045_supplementary_data.zip › zoab045-suppl_data/Supplemental_Experimental_Procedures_Eyeblink_v3.docx]

Mutual Synchronization of Eyeblinks between Dogs/Cats and Humans

**Hikari Koyasu^a, b^, Risa Goto^a^, Saho Takagi^a, b^, Miho Nagasawa^a^, Tamami Nakano^c^, Takefumi Kikusui^a,^***

^a^Laboratory of Human-Animal Interaction and Reciprocity, Azabu University, 1-17-71 Fuchinobe, Chuo-ku, Sagamihara-shi, Kanagawa 252-5201, Japan
^b^Japan Society for the Promotion of Science, 5-3-1 Kojimachi, Chiyoda-ku, Tokyo 102-0083, Japan

^c^Graduate School of Frontiers and Biosciences, Osaka University, 1-3 Yamadaoka, Suita, Osaka 565-0871, Japan

*Address correspondence to Takefumi Kikusui. Email: [kikusui@azabu-u.ac.jp](mailto:kikusui@azabu-u.ac.jp)

Handling editor: Zhi-Yun Jia (贾志云)

Received on 16 March 2021; accepted on 28 May 2021

**Keywords: blink, synchronization, communication, cat, dog**

**Supplemental Experimental Procedures**

For dogs, the tests were performed in the laboratory at Azabu University, Japan. The room was 2.5 meters × 5.0 meters, and was partitioned in half, with a dog on one side and a human on the other side. There was a 25 cm clear window in the center of the partition where humans and dogs could make eye contact. For cats, the tests took place in a room of the owner’s house. While the cat was calm in their carrier bag or in their bed, the owner or stranger sat in front of the cat and conducted the experiment. Two video cameras were used to record behavior (HX-A1H, Panasonic Corporation, Osaka, Japan; HDR-AS50, Sony Corporation, Tokyo, Japan). The sampling rate was 60 fps. Kinovea (version 0.8.15) was used for recording the blinks of humans and dogs/cats, and BORIS (version 7.10.2) was used for recording the duration that dogs and cats looked at humans. Blinks were defined as a movement of eyelids taking less than two seconds. It included incomplete blinks, such as half blinks and one-eye blinks. The number of humans acting as strangers was 27. Because the dog experiments were conducted at the laboratory, 26 college students participated as strangers. However, as the cat experiments were conducted in each owner's house, only one stranger participated. The sex of the stranger was the same as that of the owner. In the current study, the influence of sex and age of either dogs/cats or humans was not take into consideration because of small sample size.

The timing of the blinks of humans before and after the blinks of dogs/cats was analyzed. Human blink frequencies were counted three-seconds before and after the blinks of dogs/cats, using the time series of blinks for the dogs/cats as the basis for a series of three-second analysis windows (1.50 s before and 1.50 s after blink onset). A blink frequency histogram was constructed for each subject by dividing these 3 s into 12 equal windows (0.25 s) and used in the subsequent analyses. When a human blink was counted more than once in different windows, the duplicated count was adjusted by dividing each blink by the total number of blinks. Subsequently, the frequencies of human blinks divided by the times of dog and cat blinks were used to calculate the human blink frequency per dog or cat blink. A binomial test was used to determine whether the actual human blink frequency was higher than the chance level. The chance level was determined by dividing the sum of the median human blink frequencies occurring in each time window before and after the dog/cat blink by 12 (the number of time windows). Because of the repetitive analyses, the false discovery rate was adjusted using the Benjamini-Hochberg procedure. Note that we did not conduct the analysis based on the timing of human blinking because the frequency of blinks of dogs/cats (especially cats) is very different from that of humans. We analyzed the human blink frequency that occurred before the dogs/cats blinked instead.

In addition, blink rates were calculated as the frequencies of blinks per minute during the mutual gaze and one-sided gaze. The time when the dogs or cats looked at the human was defined as mutual gaze, and the time when the dogs or cats did not look at the humans was defined as the one-sided gaze of humans. Blink rate during mutual gaze was determined as the number of blinks during mutual gaze divided by the duration of mutual gaze, and blink rate during one-sided gaze was determined as the number of blinks during one-sided gaze divided by the duration of one-sided gaze. Spearman's rank correlation coefficients were determined to assess the relationship between blink rates of humans and dogs/cats. Statistical analysis software R (version 3.5.2) was used.

All (Animal and Human experiments) protocols were carried out in accordance with relevant guidelines and regulations. All experimental procedures for dogs and cats were approved by the Animal Ethics Committee of Azabu University (#180410-1). Experimental procedures for human participants were approved by the Ethical Committee for Medical and Health Research Involving Human Subjects of Azabu University (#052). All participants gave written informed consent, approved by the ethical committee, prior to the experiments.

**Table S1. Subject Information**

The information of the dogs and cats that participated in the experiment are shown. Individuals with gray shaded were excluded from the analysis.

| ID | Species | Sex | Age | Breed |  |
| --- | --- | --- | --- | --- | --- |
| D1 | Dog | Female | 6 | Mix |  |
| D2 | Dog | Male | 5 | Pomeranian | More than 30% of time was not captured human eyes |
| D3 | Dog | Male | 11 | Dalmatian |  |
| D4 | Dog | Female | 13 | Dalmatian |  |
| D5 | Dog | Female | 10 | Toy pooble | Excluded for the condition of the dog |
| D6 | Dog | Female | 10 | Shih Tzu | Human blinks are extremely low |
| D7 | Dog | Female | 5 | Border collie | More than 30% of time was not captured dog's eyes |
| D8 | Dog | Male | 3 | Border collie | More than 30% of time was not captured dog's eyes |
| D9 | Dog | Male | 12 | Shiba | No blinks during the test |
| D10 | Dog | Male | 6 | Toy pooble | Excluded for the condition of the dog |
| D11 | Dog | Female | 8 | Chihuahua | More than 30% of time was not captured human eyes |
| D12 | Dog | Female | 1 | Mix | More than 30% of time was not captured dog's eyes |
| D13 | Dog | Male | 8 | Weimaraner | More than 30% of time was not captured dog's eyes |
| D14 | Dog | Male | 4 | Golden retriever | More than 30% of time was not captured dog's eyes |
| D15 | Dog | Male | 8 | Miniature Bull Terrier | Different frame rates |
| D16 | Dog | Female | 3 | Chihuahua | More than 30% of time was not captured dog's eyes |
| D17 | Dog | Male | 9 | Toypoodle | No video of the owner |
| D18 | Dog | Male | 4 | Golden retriever | Different frame rates |
| D19 | Dog | Male | 2 | Toypoodle |  |
| D20 | Dog | Female | 1 | Toypoodle |  |
| D21 | Dog | Female | 14 | Jack russel terrier | Different frame rates |
| D22 | Dog | Female | 14 | Jack russel terrier | No video of the owner |
| D23 | Dog | Male | - | Mix | More than 30% of time was not captured dog's eyes |
| D24 | Dog | Male | - | Mix |  |
| D25 | Dog | Male | - | Mix | More than 30% of time was not captured dog's eyes |
| D26 | Dog | Male | - | Mix |  |
| C1 | Cat | Female | 10 | Mix | More than 30% of time was not captured cat's eyes |
| C2 | Cat | Male | 3 | Ragamuffin | Different frame rates |
| C3 | Cat | Female | 3 | American Shorthair |  |
| C4 | Cat | Female | 7 | Mix |  |
| C5 | Cat | Male | 9 | Mix | More than 30% of time was not captured cat's eyes |
| C6 | Cat | Male | 3 | American Curl | More than 30% of time was not captured cat's eyes |
| C7 | Cat | Male | 4 | Persian | Test interruptions due to cat conditions |
| C8 | Cat | Male | 2 | Mix |  |
| C9 | Cat | Male | 1 | Mix |  |
| C10 | Cat | Male | 1 | Mix | Test interruptions due to cat conditions |
| C11 | Cat | Male | 8 | American Shorthair | Excluded for unification of test conditions |
| C12 | Cat | Male | 5 | Ocicat | Excluded for unification of test conditions |
| C13 | Cat | Male | 20 | Mix | Test interruptions due to cat conditions |
| C14 | Cat | Female | 4 | Mix |  |
| C15 | Cat | Male | 9 | Mix |  |
| C16 | Cat | Female | 9 | Mix | More than 30% of time was not captured cat's eyes |
| C17 | Cat | Male | 15 | Russian Blue |  |
| C18 | Cat | Male | 3 | Scottish Fold |  |
| C19 | Cat | Male | 5 | Persian | Excluded for unification of test conditions |
| C20 | Cat | Male | 2 | Mix | More than 30% of time was not captured cat's eyes |
| C21 | Cat | Male | 6 | British Shorthair |  |
| C22 | Cat | Male | 3 | Mix | Test interruptions due to cat conditions |
| C23 | Cat | Female | 3 | Mix |  |
| C24 | Cat | Male | 14 | Mix | No blinks during the test |

**Table S2. Blink rates of humans, dogs and cats**

The blink rates of humans (A), dogs (B), and cats(C) are shown. The IDs correspond to the dogs and cats information in TableS1. The "With Owner/Stranger" column in the table of "Dogs’ blink rates (B)" and "Cats’ blink rates (C)" describe whether the dogs/cats participated in the test with the Owner or the Stranger.

(A) Human blink rates

| ID | Owner/Stranger | Sex | During mutual gaze | During One-sided gaze | Whole test |
| --- | --- | --- | --- | --- | --- |
| D1 | Owner | Female | 52.813 | 55.276 | 54.801 |
| D1 | Stranger | Female | 48.822 | 26.839 | 29.000 |
| D3 | Owner | Female | 35.724 | 38.588 | 36.600 |
| D3 | Stranger | Female | 81.184 | 30.041 | 50.200 |
| D4 | Owner | Female | 27.262 | 32.176 | 29.400 |
| D4 | Stranger | Female | 54.971 | 63.068 | 60.800 |
| D19 | Owner | Male | 76.035 | 29.284 | 37.400 |
| D19 | Stranger | Male | 27.790 | 33.518 | 28.200 |
| D20 | Owner | Female | 40.662 | 46.855 | 43.200 |
| D20 | Stranger | Female | 23.593 | 32.715 | 28.333 |
| D24 | Owner | Male | 28.947 | 29.014 | 29.000 |
| D24 | Stranger | Male | 25.032 | 34.119 | 32.667 |
| D26 | Owner | Female | 50.926 | 57.365 | 55.500 |
| D26 | Stranger | Female | 36.062 | 38.842 | 38.626 |
| C3 | Owner | Female | 45.167 | 40.662 | 41.400 |
| C3 | Stranger | Female | 21.333 | 24.812 | 24.420 |
| C4 | Owner | Female | 18.935 | 17.474 | 18.400 |
| C4 | Stranger | Female | 18.211 | 14.768 | 17.667 |
| C8 | Owner | Female | 10.210 | 21.979 | 20.250 |
| C8 | Stranger | Female | 14.024 | 17.848 | 17.667 |
| C9 | Owner | Female | 4.078 | 15.972 | 15.000 |
| C9 | Stranger | Female | 33.014 | 31.968 | 32.000 |
| C14 | Owner | Female | 10.993 | 20.516 | 19.000 |
| C14 | Stranger | Female | 13.386 | 10.746 | 11.667 |
| C15 | Owner | Female | 17.413 | 20.967 | 20.600 |
| C15 | Stranger | Female | 10.267 | 9.680 | 10.000 |
| C17 | Owner | Female | 20.767 | 20.260 | 20.333 |
| C17 | Stranger | Female | 15.821 | 17.445 | 17.000 |
| C18 | Owner | Female | 29.775 | 28.771 | 29.000 |
| C18 | Stranger | Female | 13.138 | 22.231 | 22.000 |
| C21 | Owner | Female | 15.348 | 27.708 | 26.500 |
| C21 | Stranger | Female | 19.183 | 10.047 | 14.333 |
| C23 | Owner | Female | 48.992 | 25.286 | 26.836 |
| C23 | Stranger | Female | 35.899 | 27.280 | 28.000 |

(B) Dogs’ blink rates

| ID | With Owner/Stranger | During mutual gaze | During one-sided gaze | Whole test |
| --- | --- | --- | --- | --- |
| D1 | Owner | 13.524 | 7.074 | 8.600 |
| D1 | Stranger | 12.027 | 3.508 | 6.200 |
| D3 | Owner | 4.625 | 6.042 | 5.000 |
| D3 | Stranger | 3.297 | 4.068 | 3.600 |
| D4 | Owner | 5.234 | 9.703 | 6.800 |
| D4 | Stranger | 10.283 | 7.060 | 8.000 |
| D19 | Owner | 2.656 | 5.075 | 3.800 |
| D19 | Stranger | 5.343 | 0.000 | 5.200 |
| D20 | Owner | 3.784 | 0.547 | 2.600 |
| D20 | Stranger | 1.157 | 0.611 | 0.800 |
| D24 | Owner | 8.575 | 9.206 | 9.000 |
| D24 | Stranger | 2.554 | 5.208 | 5.000 |
| D26 | Owner | 11.973 | 11.742 | 11.800 |
| D26 | Stranger | 13.932 | 15.400 | 15.000 |

(C) Cats’ blink rates

| ID | With Owner/Stranger | During mutual gaze | During one-sided gaze | Whole test |
| --- | --- | --- | --- | --- |
| C3 | Owner | 1.2867 | 2.3218 | 2.000 |
| C3 | Stranger | 0.0000 | 1.1685 | 1.000 |
| C4 | Owner | 7.8586 | 16.1635 | 9.400 |
| C4 | Stranger | 7.2883 | 0.0000 | 6.333 |
| C8 | Owner | 1.2110 | 1.9165 | 1.800 |
| C8 | Stranger | 19.9990 | 5.6604 | 7.333 |
| C9 | Owner | 4.0630 | 0.7471 | 1.400 |
| C9 | Stranger | 5.8519 | 2.8991 | 3.000 |
| C14 | Owner | 16.3451 | 4.1039 | 6.800 |
| C14 | Stranger | 11.9571 | 4.9203 | 7.667 |
| C15 | Owner | 9.6740 | 5.1303 | 5.600 |
| C15 | Stranger | 0.9999 | 4.0004 | 2.200 |
| C17 | Owner | 3.2874 | 2.0494 | 2.200 |
| C17 | Stranger | 1.2885 | 2.0303 | 1.800 |
| C18 | Owner | 7.2181 | 5.4199 | 6.333 |
| C18 | Stranger | 2.3177 | 0.4834 | 0.800 |
| C21 | Owner | 2.3763 | 4.4443 | 3.400 |
| C21 | Stranger | 3.1455 | 4.0705 | 3.600 |
| C23 | Owner | 13.1265 | 6.2470 | 7.400 |
| C23 | Stranger | 2.6676 | 1.9459 | 2.000 |
